# The Cap-proximal secondary structures of the 5′UTRs of parainfluenza virus 5 mRNAs specify differential sensitivity to type I interferon and IFIT1

**DOI:** 10.1099/jgv.0.002093

**Published:** 2025-03-27

**Authors:** Jacqueline Hankinson, Dan Young, Elizabeth B. Wignall-Fleming, Radoslaw Lukoszek, Victoria H. Cowling, Richard Randall, Steve Goodbourn

**Affiliations:** 1 Section for Pathogen Research, Institute for Infection and Immunity, St. George’s School of Health and Medical Sciences, City St. George’s, University of London, London, UK; 2 School of Biology, Centre for Biomolecular Sciences, University of St. Andrews, St. Andrews, UK; 3 School of Life Sciences, Centre for Gene Regulation and Expression, University of Dundee, Dundee, UK; 4 Cancer Research UK Scotland Institute, Glasglow, UK; 5 School of Cancer Science, University of Glasglow, Glasgow, UK

**Keywords:** IFIT1, IFN, mRNA Cap structure, parainfluenza virus 5 (PIV5)

## Abstract

Parainfluenza virus 5 (PIV5) is a paramyxovirus that has been isolated from numerous mammalian hosts and is notable for its ability to cause persistent infections. Although PIV5-infected cells are resistant to IFN due to the ability of the V protein to target STAT1 for degradation, PIV5 shows residual IFN sensitivity when infecting cells that have already been exposed to IFN. We have previously reported that the human IFN-stimulated gene with the greatest inhibitory effect on PIV5 is IFIT1. IFIT1 inhibits the translation of incompletely methylated mRNAs (Cap0) but not those 2′-O-methylated at the first transcribed nucleotide (Cap1). All *Mononegavirales* are thought to generate Cap1 mRNA, so the sensitivity of PIV5 to IFIT1 is surprising. Here, we show that PIV5 generates Cap0 mRNA but not Cap1 mRNA, thus explaining its sensitivity to IFIT1. Furthermore, the sensitivity of different PIV5 genes to IFIT1-mediated translation inhibition varies. In the absence of complete Cap methylation, we show that the presence or absence of 5′-terminal RNA hairpin structures in the 5′UTRs of PIV5 genes determines the extent to which they are sensitive to IFIT1. Notably, the genes involved in RNA synthesis are relatively resistant to IFIT1 inhibition. This presents a potential mechanism by which IFIT1 can regulate the outcome of PIV5 infection in response to IFN and may be important in allowing the virus to establish prolonged/persistent infections.

## Introduction

Parainfluenza virus 5 (PIV5) is a *Rubulavirus* and a member of the *Paramyxoviridae*, a family of single-stranded negative-sense RNA viruses [1]. PIV5 is able to infect a wide range of mammalian hosts, including humans, and has been linked to disease in dogs, cattle and pigs [2,6]. Research on this virus has helped to set paradigms for how RNA viruses evade the cellular IFN response [7,8], and more recently, PIV5 has served as a model of RNA virus persistent infection [9]. PIV5 is able to persistently infect cell lines with minimal cytopathic effect [10,12] and has been isolated at late times post-infection from both dogs [13] and humans [14,16].

PIV5 has a genome of 15,246 nucleotides with six genes that encode seven proteins [nucleoprotein (NP), V/P, matrix (M), fusion (F), haemagglutinin-neuraminidase (HN) and large (L), with a seventh gene, small hydrophobic (SH), being present in some strains], which are transcribed into separate mRNAs, each of which is capped at the 5′ end, polyadenylated at the 3′ end and has both 5′ and 3′UTRs [17]. The viral polymerase is formed of the L protein in complex with the phosphoprotein (P), which recognizes the nucleocapsid, a structure in which the viral genome is encapsidated by viral NP. The L protein is multi-functional, performing RNA replication, transcription and mRNA capping/methylation. The M protein coordinates virion assembly by interacting with both the nucleocapsid and the cytoplasmic tails of the membrane glycoproteins HN and F, which, respectively, mediate attachment to and entry of the virion into cells.

In addition to the aforementioned essential proteins, PIV5 encodes the V protein which shares an N-terminus with P but differs at the C-terminus due to the insertion of non-templated guanosine nucleotides during transcription of the V/P gene in a process termed ‘mRNA editing’. The V protein is an accessory protein with a major function being the evasion of the host cell IFN response [8], although the V protein may also play a role in regulating viral RNA synthesis [18,19]. The PIV5 V protein limits the induction of type I IFNs by inhibiting the activation of both the RNA pattern recognition receptors, mda5 and RIG-I [20,22]. The V protein of PIV5 also disables the cellular signalling response to type I IFN by targeting Signal Transducer and Activator of Transcription (STAT) 1 for ubiquitin-mediated proteasomal degradation by binding to the STAT2 component of the STAT1–STAT2 heterodimer and recruiting cellular ubiquitination machinery that ubiquitinates STAT1 (reviewed in reference [8]). Degradation of STAT1 disrupts the Janus Kinase (JAK)-STAT signalling pathway downstream of IFN binding to the IFN receptor and prevents expression of IFN-stimulated genes (ISGs) in cells infected by PIV5 [23].

Despite the efficient disruption of JAK-STAT signalling by the V protein, PIV5 is still subject to the action of ISGs when infecting cells that have already been exposed to IFN, at least until the virus has dismantled the antiviral state by degrading STAT1. We have previously identified IFIT1 as an ISG with powerful antiviral activity against PIV5 [24,25]. The role of IFIT1 is to strongly attenuate the translation of target mRNAs. All eukaryotic cellular mRNAs are capped at their 5′ end with an inverted N7-methylated guanosine (m7G) linked to the first transcribed nucleotide by a 5′−5′ triphosphate bridge [26,27] (this is referred to as ‘Cap0’). Importantly, cellular Cap structures are further modified at the 2′ hydroxyl of the ribose of the first transcribed nucleotide by Cap methyltransferase (CMTR) 1 to form a ‘Cap1’ structure [28,29], and a portion of transcripts is then further 2′-O-methylated at the ribose of the second transcribed nucleotide by CMTR2 to form a ‘Cap2’ structure [30]. The m7G Cap is necessary for mRNA stability [31], splicing, nuclear export and efficient translation [32], and as discussed below, the additional Cap methylations play a role in distinguishing ‘self’ from ‘non-self’ mRNAs [33]. IFIT1 has a high affinity for Cap0 mRNA and is able to outcompete the binding of the translation factor eIF4E, thus preventing the translation of Cap0 transcripts [34,35]. IFIT1 has a much reduced affinity for Cap1 structures and, thus, is able to discriminate between mRNAs based on the presence or absence of 2′-O-methylation of the first transcribed nucleotide [34,37].

To evade IFIT1, most mammalian viruses have evolved mechanisms to generate Cap1 mRNA such as the encoding of viral 2′-O-methyltransferases (2′-O-MTases) [38,40] or ‘Cap-snatching’ from cellular transcripts [41,42]. Paramyxoviruses, including PIV5, are predicted to encode a 2′-O-MTase within domain VI of the L protein [38] and would therefore be predicted to produce Cap1 structures, which prevent binding of IFIT1. The sensitivity of PIV5 to IFIT1 is therefore unexpected. We have previously shown [25] that PIV5 mRNAs extracted from infected cells show different sensitivities to IFIT1 in *in vitro* translation experiments; the translation of mRNA for the NP and P proteins, which are essential for viral replication, shows some resistance to IFIT1, whilst the translation of the mRNA for the M protein is strongly inhibited by IFIT1. Some additional degree of IFIT1-resistance can be conferred upon NP and P, but not M, mRNAs by forcing Cap1 modification *in vitro* using a cellular enzyme, suggesting that there may be inefficient 2′-O-methylation of transcripts [25]. Here, we show that PIV5 mRNAs appear to lack the Cap1 structure. Whilst it would be predicted that the lack of Cap1 modification would render all PIV5 transcripts sensitive to IFIT1, we show that the relative insensitivity of some viral mRNAs is a consequence of them having a clear Cap-proximal RNA secondary structure, which is predicted to interfere with IFIT1 binding. We propose that the virus has evolved differing sensitivity of viral mRNAs to IFIT1 as a mechanism to regulate viral protein production in response to IFN and may be important in allowing the virus to establish prolonged/persistent infections.

## Methods

### Cells, viruses and infections

A549 (ATCC CCL-185), A549.GFP (ref [43]), 293 (ATCC CRL-1573), Vero (ATCC CCL-81) and BSRT7 (BHK-21 cells stably expressing T7 RNA polymerase [44]) cells were grown at 37 °C and 10% CO_2_ and maintained in Dulbecco’s Modified Eagle Medium (DMEM) supplemented with 100 U ml^−1^ penicillin, 100 µg ml^−1^ streptomycin and 10% FBS.

The pBH276 plasmid (a kind gift from Professor Robert Lamb, Northwestern University) containing the full-length genome sequence of the W3A strain of PIV5 (accession JQ743318), a variant containing a serine to phenylalanine substitution at aa 157 (F157) and the construction of PIV5 with an extra gene encoding mCherry between the HN and L genes have been previously described [45,46]. Mutations to the M 5′UTR were introduced into the F157 backbone by Gibson Assembly; these changes to the PIV5 genome were A^3123^G, C^3124^G, A^3125^G and U^3127^A. Viruses were rescued by co-transfection of BSRT7 cells with pCAGGS plasmids expressing the NP, P and L genes of PIV5 together with the full-length genome plasmid (pBH276), which contains a T7 promoter. Per well of a six-well plate seeded with BSRT7 at 50–70% confluence, 1,000 ng pBH276, 100 ng pCAGGS-NP, 100 ng pCAGGS-P(CPI+) and 500 ng pCAGGS-L were transfected using Lipofectamine LTX/Plus (Thermo Fisher). At 2 days post-transfection, the medium was replaced with DMEM with 2% FBS, and cells were incubated at 37 °C for 5–6 days. The supernatant was then harvested, and a 20 µl aliquot was used to infect near-confluent Vero cells in a T75 flask in DMEM with 2% FBS. At five dpi, the supernatant was aliquoted to microcentrifuge tubes, flash-frozen in liquid nitrogen and stored at −80 °C. Virus stocks and samples were titrated by plaque assay in Vero cells.

Type I IFN (Intron A: Schering-Plough) was stored at −80 °C at 10^6^ IU ml^−1^ and used at a final concentration of 1,000 IU ml^−1^.

### Minigenome assay

The PIV5 minigenome plasmid encoding firefly luciferase (pPIV5MG-Fluc.ter) has been described previously [9]. For each well of a 12-well plate seeded with subconfluent 293 IFIT1^-/-^ cells, the following amounts of plasmid were transfected using linear polyethyleneimine (PEI) of molecular weight 25,000 (Polysciences Inc., Warrington PA, USA) under standard conditions: 8 ng minigenome plasmid, 30 ng pCAGGSV5-NP, 30 ng pCAGGSV5-CPI+-P, 150 ng pCAGGSV5-L, 150 ng pCAGGSV5-T7pol and 32 ng pCAT-LAC (a *β*-galactosidase-expressing transfection control plasmid). The total amount of DNA was made up to 650 ng using either empty pCAGGSV5 or pEFV5. One hundred twenty-five nanograms of pEFV5 expressing either hIFIT1 (NP_001539.3) or mIFIT1 (NP_032357.2) were co-transfected as indicated in the Results section. At 48 h post-transfection, firefly luciferase and *β*-galactosidase activity were assayed and normalized as described previously [9].

For the measles virus (MV) minigenome assay, a reporter was generated in which the PIV5 trailer and leader sequences of pPIV5MG-Fluc.ter were replaced by the trailer and leader sequences of the MV Edmonton strain (pMVMG-Fluc.ter). cDNAs for the ORFs of the NP, P and L proteins of MV were generated by PCR from plasmids provided by Professor Paul Duprex (University of Pittsburgh), cloned into the eukaryotic expression vector, pCAGGS, and sequence verified using Sanger sequencing (Source BioScience, UK). The MV minigenome transfection and assay conditions were as described above for PIV5.

The bicistronic PIV5 minigenome was adapted from the standard PIV5 minigenome described above. The non-coding termini on either side of the firefly luciferase CDS in the original minigenome are maintained in the bicistronic minigenome. Between these are, in sequence, the Renilla luciferase CDS, the full-length NP 3′UTR of PIV5, the NP/P intergenic sequence, a variable 40-nucleotide sequence, e.g. the first 40 bases of the M 5′UTR, and the CDS of firefly luciferase. Transfection and firefly luciferase assay conditions were as for the standard PIV5 minigenome.

### Clustered regularly interspaced short pallindromic repeats (CRISPR)-Cas9-mediated gene knockout

CRISPR-Cas9 sgRNAs were expressed from the ‘double nickase’ paired plasmids, pSpCas9(BB)−2A-GFP (pX461: a kind gift from Feng Zhang; Addgene plasmid #48140; http://n2t.net/addgene:48140; RRID:Addgene_48140) and pSpCas9(BB)−2A-Puro (pX462) V2.0 (a kind gift from Feng Zhang; Addgene plasmid #62987; http://n2t.net/addgene:62987; RRID:Addgene_62987) [47]. The binding sites of the sgRNAs used for the knockout of hIFIT1 were GAATGAGGAAGCCCTGAAGA and AGAAGCTGAAAACTTAATGC; the second site is given as the top strand sequence despite the sgRNA being the reverse complement. Sub-confluent A549.GFP or 293 cells in a six-well plate were transfected with 500 ng of each plasmid per well using PEI. After 24 h, transfected cells were selected by puromycin (2 µg ml^−1^ for 293 cells and 4 µg ml^−1^ for A549 cells), and single clones were expanded and screened for successful knockout of IFIT1 by Western blot and DNA sequencing.

### SDS-PAGE and immunoblotting

Collected cells were resuspended in lysis buffer (150 mM NaCl, 50 mM Tris-HCl pH 7.4, 1 mM EDTA and 1% Triton X-100) with added protease inhibitors (1 mM benzamidine, 30 µg ml^−1^ leupeptin, 5 µg ml^−1^ aprotinin and 5 µg ml^−1^ pepstatin A). An equal amount of protein lysate was separated by 10% SDS-PAGE. Following transfer to a PVDF membrane (Immobilon), proteins were detected using mouse anti-NP (1 : 1,500), mouse anti-P/V (1 : 2,000), mouse anti-M (1 : 1,000) (all described in reference [48]), rabbit anti-hIFIT1 (Thermo Fisher, PA5-27907; 1 : 1,000), rabbit anti-tubulin (Abcam, ab176560; 1 : 1,000) or mouse anti-tubulin (Sigma, T9026; 1 : 2,500). Secondary antibodies (Licor IRDye 680RD goat anti-rabbit and Licor IRDye 800CW goat anti-mouse) were both used at 1 : 15,000.

### Protein sequence alignment

L protein MTase domains were aligned using Clustal Omega (1.2.4). Accession numbers for each virus L protein are as follows: canine distemper virus (NP_047207.1), MV (NP_056924.1), Sendai virus (NP_056879.1), parainfluenza virus 3 (NP_067153.2), Hendra virus (NP_047113.3), Nipah virus (NP_112028.1), PIV5 (YP_138518.1) and mumps virus (MuV) (NP_054714.1).

### Cap analysis protocol with minimal analyte processing (CAP-MAP) analysis

A549 cells were either mock-infected, IFN-treated or infected with PIV5/F157 at an moi of 10. At 24 hpi, cells were harvested, and mRNA was isolated from total RNA. Following digestion by nuclease P1, Cap dinucleotides were resolved by liquid chromatography (LC) and identified by mass spectroscopy, as detailed in [49].

### RNA structure predictions

Predictions were made using the RNAfold WebServer (http://rna.tbi.univie.ac.at//cgi-bin/RNAWebSuite/RNAfold.cgi) with the default parameters.

### Protein purification

Plasmids encoding recombinant N-terminally His6-tagged hIFIT1 or mIFIT1 capable of being expressed in *Escherichia coli* were generated by inserting the cDNAs for hIFIT1 or mIFIT1 in-frame between the NcoI and EcoRI sites of the pET His6 Sumo TEV LIC cloning vector (a kind gift from Scott Gradia, University of California, Berkeley: Addgene plasmid #29711; http://n2t.net/addgene:29711; RRID:Addgene_29711). Proteins were expressed in and purified from *E. coli* strain BL21(DE3). Transformed cells were grown at 37 °C until an OD600 of around 0.6 was reached, at which point protein expression was induced using 50 µM IPTG and then incubated for a further 5 h. Cells were lysed in lysis buffer (50 mM sodium phosphate, 300 mM NaCl, 0.05% v/v Tween 20, 10 mM imidazole and pH 8.0) containing lysozyme and protease inhibitors (1 mM benzamidine, 30 µg ml^−1^ leupeptin, 5 µg ml^−1^ aprotinin and 5 µg ml^−1^ pepstatin A). Following sonication, samples were centrifuged at 50,000 ***g*** for 20 min at 4 °C. The lysate supernatant was added to an equilibrated slurry of Pierce Ni-NTA Magnetic Agarose Beads (Thermo Fisher) and mixed for 1 h at 4 °C. The beads were collected and washed with 1 ml wash buffer (50 mM sodium phosphate, 300 mM NaCl, 0.05% v/v Tween 20, 15 mM imidazole and pH 8.0) followed by a second wash with a higher imidazole concentration (50 mM). Washed beads were resuspended in 1 ml of elution buffer (50 mM sodium phosphate, 300 mM NaCl, 0.05% v/v Tween 20, 300 mM imidazole and pH 8.0), and 6 ml of desalting elution buffer (20 mM Tris pH 8.0, 150 mM NaCl, 1 mM DTT and 5% v/v glycerol) was added. The sample was centrifuged at 4,000 ***g*** for 20 min at 4 °C through an Amicon Ultra-15 Centrifugal Filter Unit (Merck) with a 30 KDa cut-off. Eight millilitres of desalting elution buffer were added, and the filter units were centrifuged again. The remaining liquid above the filter was increased in volume to about 1 ml, aliquoted and stored at −80 °C. Protein concentration was determined by Bradford assay.

### *In vitro* transcription and RNA probe labelling

*In vitro* transcription was carried out using a HiScribe T7 High Yield RNA Synthesis Kit (New England Biolabs) with 1 U µl^−1^ RiboLock RNase Inhibitor (Thermo Fisher). DNA templates containing a class II T7 promoter and the first 40 nucleotide of each PIV5 5′UTR (with an additional 3′ AAAA to increase 3′-labelling efficiency) were generated by PCR, gel-purified, with elution into water. The concentration of m7GpppA Cap analogue (New England Biolabs, S1405) in each *in vitro* transcription reaction was 8 mM, and ATP was 2 mM. Reactions were left for 24 h at 37 °C before being fractionated by electrophoresis on a denaturing 14% 19 : 1 acrylamide:bisacrylamide gel to separate capped from uncapped RNA products. The gel was then stained with methylene blue staining solution (1 : 1 0.4M NaOAc:0.4M acetic acid and 0.2% w/v methylene blue) for 15 min and destained with water until bands were clearly visible. RNA was eluted from the gel in RNA gel elution buffer (20 mM Tris–HCl pH 7.5, 0.25M NaOAc, 1 mM EDTA pH 8.0 and 0.25% w/v SDS). The supernatant was transferred to a fresh tube, and RNA was extracted with an equal volume of water-saturated phenol. Following centrifugation, the aqueous phase was transferred to a fresh tube and purified by Monarch RNA Cleanup kit (New England Biolabs). The 3′-labelling of RNA with pCp-Cy5 (Jena Bioscience, NU-1706-CY5) was performed with 0.5 units per microlitre T4 RNA ligase (New England Biolabs), 1 mM ATP, 1x T4 reaction buffer, 15% DMSO and 2 µM pCp-Cy5 at 16 °C for 16 h followed by 1 h at 37 °C, all in the dark. Following purification by the Monarch RNA Cleanup kit, RNA was quantified and diluted to 100 nM in water. All enzymatic reactions were carried out in the presence of 1 U µl^−1^ RiboLock RNase Inhibitor.

### Electrophoretic mobility shift assay

Binding reactions were set up in a volume of 10 µl containing a final concentration of 40 nM RNA, 0.4 U µl^−1^ RiboLock, 2 µl 5× binding buffer (75 mM HEPES pH 7.4, 50 mM KCl, 1 mM DTT, 25 mM MgCl_2_ and 50% v/v glycerol), 0.01 mg ml^−1^ BSA and purified IFIT1 protein diluted in protein elution buffer (see protein purification section). Prior to the addition of recombinant IFIT1 protein, samples were left at room temperature for 15 min to encourage RNA folding. Upon addition of IFIT1, samples were left for another 30 min at room temperature to allow protein/RNA binding. Each sample was loaded (without loading dye) into an 8% native gel (Accugel 40% acrylamide:bisacrylamide 29 : 1, National Diagnostics) made with LAB buffer (10 mM LiOAc and 10 mM boric acid). The running buffer (LAB) was pre-chilled to 4 °C, and the gel was run at 250 V for 45 min. The gel was imaged on a ChemiDoc MP machine (Bio-Rad) using the default settings for Cy5.

### Data presentation and analysis

Growth curves, histograms and statistical analyses (paired t-test) were prepared using GraphPad Prism software (v10.2.1). All experiments were carried out at least three times, and error bars are shown as the sem. Where indicated in figures, a *P* value of <0.05 is indicated by ^∗^, a *P* value of <0.01 is indicated by ^∗∗^ and a *P* value of <0.001 is indicated by ^∗∗∗^.

## Results

**Fig. 1.**
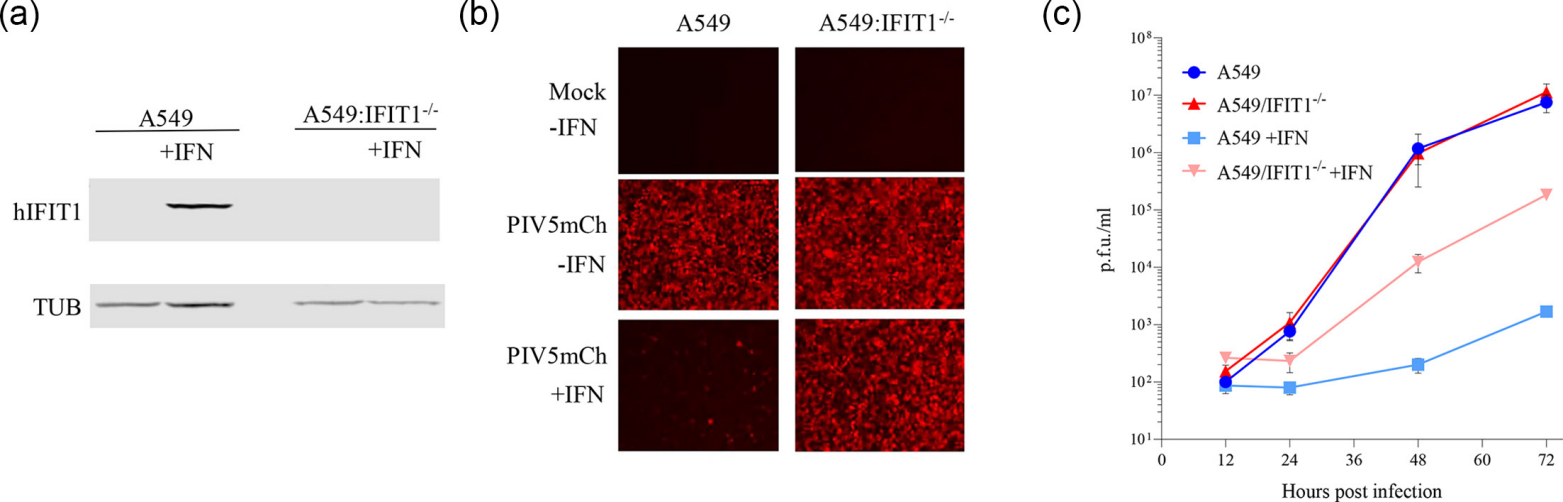
IFIT1 is the main ISG responsible for inhibition of PIV5 translation. (**a**) A549 and A549 IFIT1^-/-^ cells were either untreated or treated with IFN and processed for immunoblotting at 18 h post-treatment. Blots were probed with antibodies against human IFIT1 and against tubulin (TUB). (**b**) A549 and A549 IFIT1^-/-^ cells were either untreated or treated with IFN for 8 h and then infected with PIV5/F157/mCherry at an moi of 10. At 18 hpi, images were taken with a fluorescent microscope. (**c**) Growth curves of PIV5/F157/mCherry in both A549 and A549 IFIT1^-/-^ cells, with or without pre-treatment with IFN for 8 h. Cells were infected at an moi of 0.01, samples were collected at the indicated timepoints and virus titre was determined by plaque assay on Vero cells.

Although the V protein of PIV5 dismantles IFN signalling by efficiently targeting STAT1 for degradation [7,23], the virus will still be exposed to ISGs upon infection of a cell already in an antiviral state. We have previously reported that the human ISG primarily responsible for the sensitivity of PIV5 to IFN is IFIT1 [24,25]. Our previous observations utilized A549 cells expressing an shRNA directed against IFIT1; to confirm the importance of IFIT1 in PIV5 infection, we used CRISPR-Cas9 editing to knock out IFIT1 in A549 cells (Fig. 1a) and infected either untreated or IFN-pre-treated cells with PIV5/F157/mCherry, which expresses mCherry from an inserted gene between HN and L [46] and which has an S157F change in the P protein that increases the rate of viral RNA synthesis [9]. Upon infection of IFN-pre-treated A549 cells, very few cells express detectable mCherry, but knockout of IFIT1 is sufficient to restore viral gene expression to levels seen in the absence of IFN (Fig. 1b). To investigate the impact of IFIT1 on the whole PIV5 life cycle, we infected A549 and A549 IFIT1^-/-^ cells and measured virus production between 12 and 72 hpi (Fig. 1c). In untreated cells, knockout of IFIT1 had no impact on viral growth. However, in IFN-pre-treated cells, knockout of IFIT1 increased viral titre at 48 and 72 hpi by about 100-fold relative to A549 WT cells. Unlike the complete rescue of viral gene expression seen in Fig. 1(b), knockout of IFIT1 was not sufficient to fully rescue viral titres to those seen in untreated A549 cells, suggesting that ISGs other than IFIT1 inhibit various other stages of the PIV5 life cycle as previously suggested [24].

**Fig. 2.**
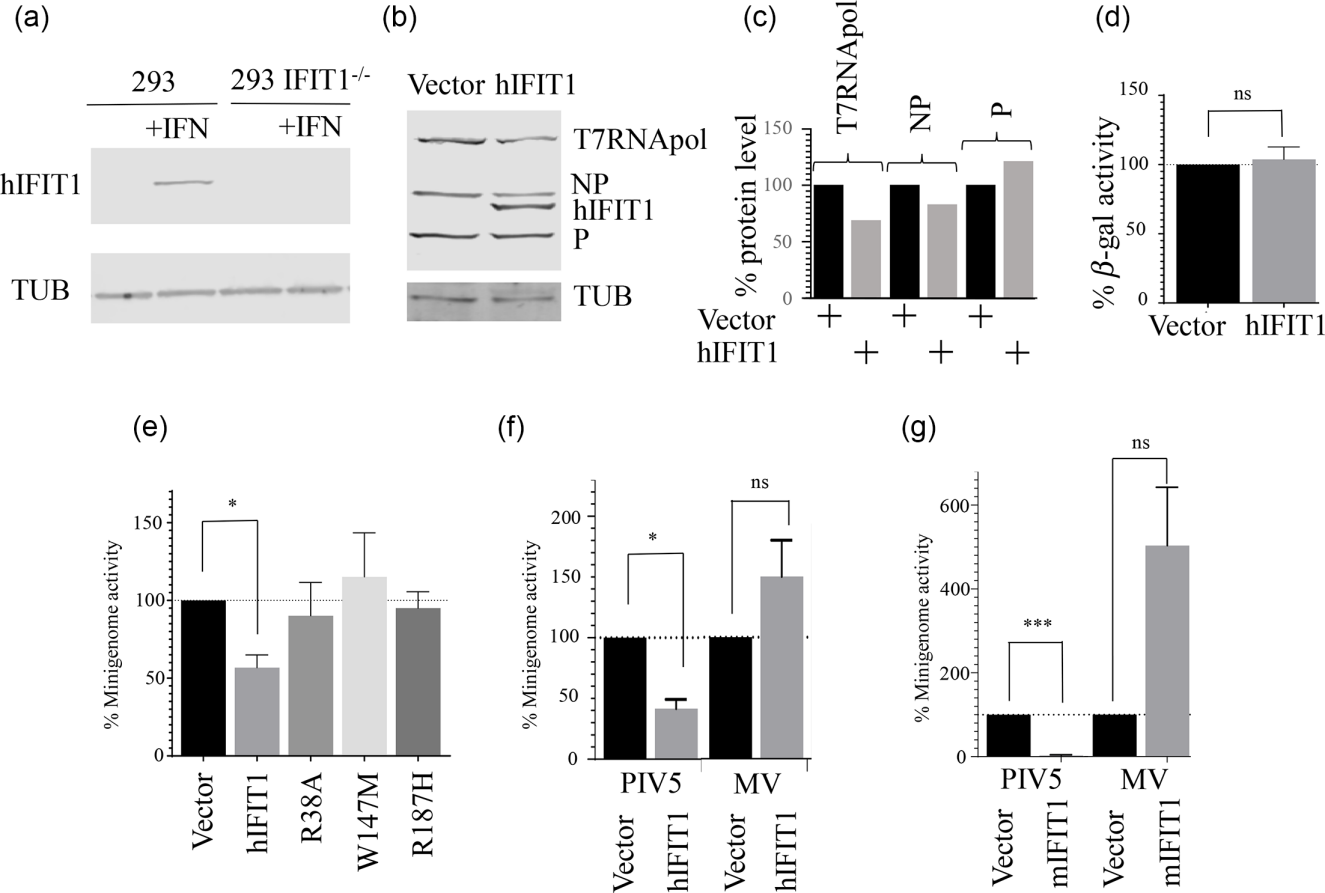
IFIT1 inhibits the PIV5 minigenome but not the MV minigenome. (a) 293 and 293 IFIT1^-/-^ cells were either untreated or treated with IFN and processed for immunoblotting at 18 h post-treatment. Blots were probed with antibodies against human IFIT1 and against tubulin (TUB). (**b**) The V5-tagged PIV5 NP and P helper plasmids and a plasmid expressing V5-tagged T7 RNA polymerase were co-transfected with either pEF empty vector or pEFV5-hIFIT1 and analysed by Western blotting for V5 or TUB. (**c**) Densitometric analysis of the Western blot data in panel (b); protein levels are normalized to the tubulin signal for each sample. (**d**) A plasmid expressing *β*-galactosidase was transfected with or without pEF-IFIT1 and *β*-galactosidase activity was assayed at 48 h post-transfection. (**e**) PIV5 minigenome activity was assayed from cells co-transfected with pEF-hIFIT1 or the indicated hIFIT1 mutant. (**f, g**) PIV5 and MV minigenome activity were assayed with or without co-transfected human (**f**) or murine (**g**) IFIT1.

Minigenome reporter systems recapitulate viral RNA synthesis and translation and are therefore a useful tool to study these processes. To test whether the PIV5 minigenome system can be used to investigate the specific impact of IFIT1 on viral gene expression, we first generated 293 IFIT1 knockout cells using CRISPR/Cas9 editing (Fig. 2a). These cells were then transfected with a plasmid expressing hIFIT1 or the ‘empty’ vector and were also transfected with the PIV5 minigenome system – the latter comprises a luciferase reporter under the control of the NP 5′UTR and the viral leader and trailer regions, together with expression vectors allowing the synthesis of codon-optimized T7 RNA polymerase, and the transcription and replication PIV5 ‘helper’ proteins, NP, P and L. An important feature of these experiments is that (because they are generated by host transcriptional machinery in the nucleus) the mRNAs for each of IFIT1, T7 RNA polymerase and the PIV5-NP, PIV5-P and PIV5-L proteins are predicted to have Cap1/Cap2 structures and, therefore, be IFIT1-resistant.

We first determined that the levels of PIV5 NP and P were not affected by the expression of IFIT1 (Fig. 2b, c). This was a potential concern because IFIT1 has been reported to globally affect translation by binding to eIF3 [50,51], but here, no notable effect on the translation of plasmid-derived transcripts was apparent. Additionally, the expression of *β*-galactosidase from an actin promoter and 5′UTR was not significantly affected by hIFIT1 (Fig. 2d).

Luciferase expression from the PIV5 minigenome system was robust and dependent upon all three PIV5 helper proteins [9]. Co-transfection of hIFIT1 with the PIV5 minigenome significantly inhibited minigenome activity, confirming that PIV5 replication and/or translation is sensitive to hIFIT1 (Fig. 2e). However, the introduction into hIFIT1 of mutations known to affect its ability to bind the mRNA Cap [36,37, 52] abrogated its ability to restrict the PIV5 minigenome, showing that the inhibitory effect of hIFIT1 is specific to its ability to bind the mRNA Cap structure and inhibit the translation of PIV5 transcripts. It should be noted that the luciferase transcript in these experiments has a 5′UTR that is derived from the NP gene; although we will discuss below that the 5′UTR of NP shows some insensitivity to IFIT1, this is incomplete, and we assume that the inhibition seen in this experiment is a consequence of the overexpression of IFIT1. We and others have previously reported that sensitivity to IFIT1 may be unique to the *Rubulavirus* genus and that other members of *Paramyxoviridae* and *Mononegavirales* are not sensitive [25,53]. Using a measles virus (MV) minigenome system as an example of a non-*Rubulavirus* paramyxovirus, we compared the sensitivity of the PIV5 and MV minigenomes to human and murine IFIT1 (mIFIT1) proteins (Fig. 2f, g, respectively). In contrast to PIV5, MV minigenome translation was found to not be inhibited by hIFIT1 and was instead increased for unknown reasons. mIFIT1 is a paralog of hIFIT1 [54], that has been well-characterized as an mRNA Cap-binding protein with a higher affinity for Cap0 than hIFIT1 [35,55]. PIV5 was extremely sensitive to mIFIT1 with near-total inhibition of the minigenome signal. In contrast, MV was not inhibited by mIFIT1 and instead displayed the IFIT1-dependent increase in signal previously seen with hIFIT1. Together, these data confirm that PIV5 is highly sensitive to translational inhibition by IFIT1 and that this property likely does not apply to paramyxoviruses outside the *Rubulavirus* genus, such as MV.

**Fig. 3.**
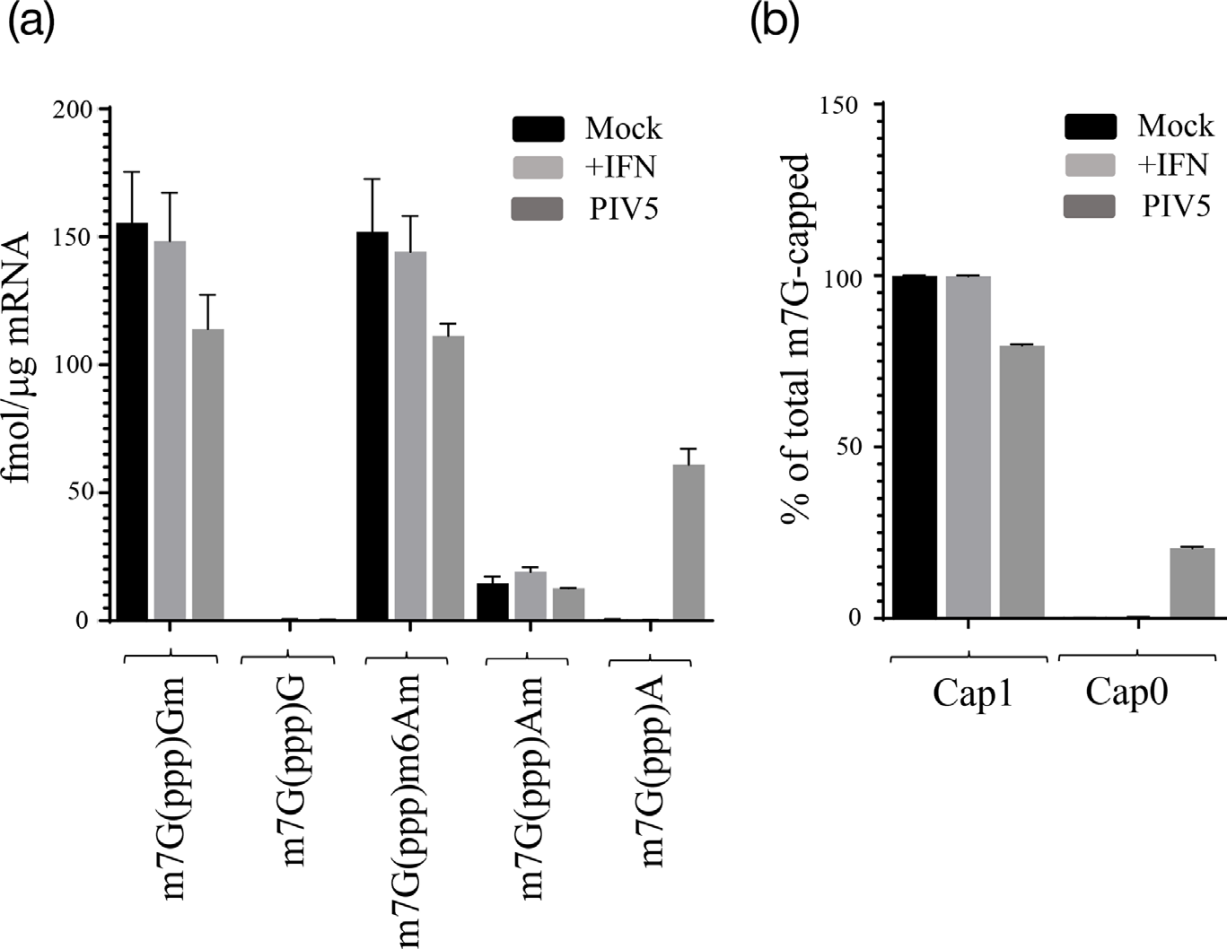
CAP-MAP analysis reveals that PIV5 mRNAs bear unmethylated Cap0 structure. A549 cells were either mock-infected, treated with IFN or infected with PIV5/F157 at an moi of 10. At 24 hpi, mRNA was isolated and digested with nuclease P1, and the structure of the resulting Cap dinucleotides was determined by LC-MS (‘CAP-MAP’ analysis). Results are presented as (**a**) the amount of each Cap dinucleotide in femtomoles per microgram of mRNA and (**b**) the proportion of each Cap structure (Cap1 or Cap0) in each condition.

Based on the observations that we were able to render the translation of some PIV5 mRNAs partially resistant to IFIT1 by forced generation of Cap1 structures [25] and that PIV5 gene expression shows differential sensitivity to IFN and IFIT1 (NP, P and L are partially resistant to downregulation in response to IFN, whereas M and HN are completely sensitive – we lacked suitable antibodies to test the F or SH proteins [24,25, 56]), we previously hypothesized that the different PIV5 transcripts showed transcript-specific differences in the extent of their Cap modification (i.e. some of the relatively insensitive transcripts, NP, P and L, may have some Cap1 structures [25]). To investigate this possibility, we employed CAP-MAP analysis in which cellular transcripts are digested by nuclease P1 to generate Cap dinucleotides, the structures of which are then quantitated by LC-MS [49] (Fig. 3). In uninfected A549 cells, either untreated or treated with IFN, mRNAs with Cap0 were undetectable, and all transcripts bore Cap1 at minimum (Cap2, generated by the additional methylation of the ribose of the second transcribed nucleotide, cannot be detected by CAP-MAP). In contrast, in cells infected with PIV5, ~20% of all transcripts bore the Cap0 structure. All such Cap0 dinucleotides were m7GpppA, and since all PIV5 transcripts begin with an adenosine, these are almost certainly all of viral origin. We have previously reported that in cells infected with PIV5 containing the S157F change in the P protein (used in these experiments because of the high level of viral RNA synthesis compared to the lab-adapted W3A strain [9]), viral transcripts account for 10–20% of the total mRNA [57]. Therefore, the data shown in Fig. 3 suggest that most, if not all, of the mRNA generated by PIV5 during infection bears the Cap0 structure.

**Fig. 4.**
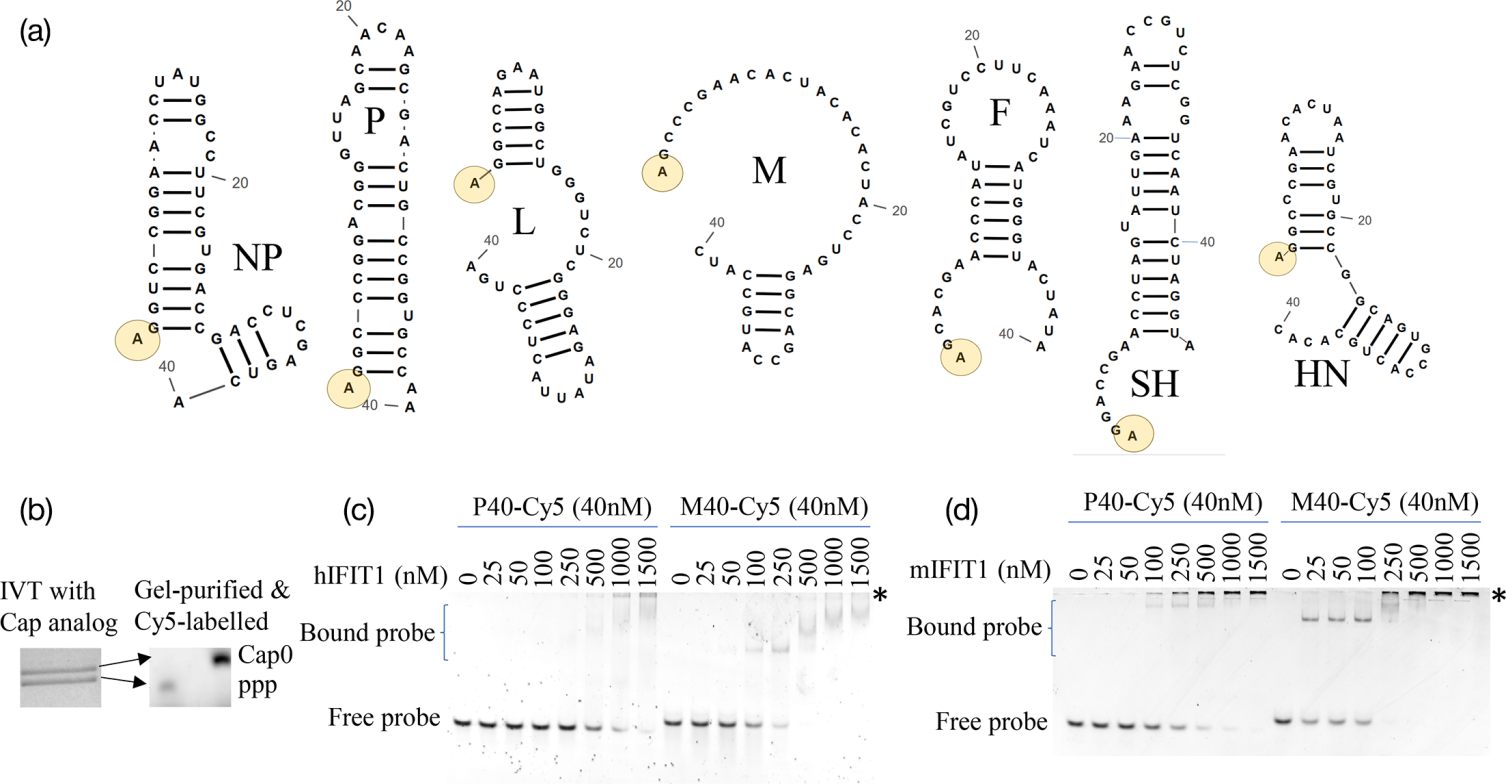
Lack of predicted RNA structure of PIV5 mRNA correlates with affinity for IFIT1. (**a**) Predicted RNA secondary structures of the first 40 bases (46 bases for SH) of the mRNAs of PIV5. The 5′ end is indicated by a yellow circle. (**b**) The first 40 bases of either the P (**P40**) or M (**M40**) genes were *in vitro* transcribed in the presence of an m7GpppA Cap analogue. The capped product was gel-purified and 3′-labelled with Cy5. (**c, d**) Electrophoretic mobility shift assays of PIV5 5′UTRs and IFIT1. Each labelled RNA probe was re-heated and added to a binding reaction at a final concentration of 40 nM along with the indicated concentration of purified recombinant hIFIT1 (**c**) or mIFIT1 (**d**) before being resolved on a non-denaturing gel. The running positions of the unbound free probe and the shifted bound complexes are indicated; complexes retained at the loading origin of the gel are marked with an asterisk.

Assuming that all PIV5 transcripts are Cap0, it is difficult to account for our previously observed differences in sensitivity to IFIT1 (for example, whilst the M protein is nearly undetectable in the presence of IFIT1, the levels of the NP and P proteins are reduced by only about 50% [24,25, 56]). We hypothesized that differential IFIT1 sensitivity might arise from differences in the sequence or structures of the different 5′UTRs. It should be noted that the presence or absence of an RNA hairpin structure at the 5′ end of different Venezuelan equine encephalitis virus (VEEV) strains determines their relative sensitivity to IFIT1 [58]. With this in mind, we analysed the predicted structures of the first 40 bases of each of the PIV5 transcripts. Fig. 4(a) shows that each of the relatively IFIT1-resistant mRNAs (NP and P) is predicted to have a single unpaired base at the 5′ end followed by an immediate region of hairpin secondary structure of at least 5 bp; although we have not analysed the IFIT1 sensitivity of the L mRNA, we note that it also has this structure. Of the IFN-sensitive mRNAs, only the 5′UTR of HN had any Cap-proximal secondary structure, with a 6 bp stem interrupted by a single nucleotide bulge – all of the other mRNAs have disordered 5′UTRs.

In order to experimentally confirm the link between sensitivity to IFIT1 and the differing predicted RNA structure of each PIV5 5′UTR, we chose to analyse the P and M 5′UTRs as respective exemplars of relatively IFIT1-insensitive and IFIT1-sensitive mRNAs. We generated fluorescently labelled RNA probes consisting of the first 40 bases of the P and M 5′UTRs (P40 and M40) with a 5′ Cap0 structure (Fig. 4b). *In vitro* transcription of probes was carried out in the presence of the m7GpppA Cap analogue, and capped probes were gel-purified to separate them from uncapped. The interaction between the RNA probes and purified recombinant hIFIT1 and mIFIT1 was then evaluated by an electrophoretic mobility shift assay. The M40 probe was found to have a much greater affinity for both hIFIT1 and mIFIT1 than the P40 probe (Fig. 4c, d). Whilst shifted complexes were visible for M40 at concentrations of 100 nM (for hIFIT1) and 25 nM (for mIFIT1), P40 required fourfold or fivefold greater protein concentrations to be shifted. Interestingly, the binding of mIFIT1 and hIFIT1 to the M40 RNA appears to generate more than one complex and generates complexes that are retained at the loading origin with increasing protein concentration; we speculate that these complexes represent the binding of multiple copies of IFIT1, with the binding perhaps being cooperative – the oligomerization of IFIT proteins has been characterized by other groups (reviewed in reference [59]). Even at the highest concentration of IFIT1 used here – 1,500 nM, the estimated peak cytosolic concentration of IFIT1 during a cellular antiviral response [60] – some amount of the P40 probe remained unbound, whereas the M40 probe was fully bound at concentrations well below 1,000 nM.

In order to experimentally confirm the link between sensitivity to IFIT1 and the differing predicted RNA structure of each PIV5 5′UTR, we chose to analyse the P and M 5′UTRs as respective exemplars of relatively IFIT1-insensitive and IFIT1-sensitive mRNAs. We generated fluorescently labelled RNA probes consisting of the first 40 bases of the P and M 5′UTRs (P40 and M40) with a 5′ Cap0 structure (Fig. 4b). *In vitro* transcription of probes was carried out in the presence of the m7GpppA Cap analogue, and capped probes were gel-purified to separate them from uncapped. The interaction between the RNA probes and purified recombinant hIFIT1 and mIFIT1 was then evaluated by an electrophoretic mobility shift assay. The M40 probe was found to have a much greater affinity for both hIFIT1 and mIFIT1 than the P40 probe (Fig. 4c, d). Whilst shifted complexes were visible for M40 at concentrations of 100 nM (for hIFIT1) and 25 nM (for mIFIT1), P40 required fourfold or fivefold greater protein concentrations to be shifted. Interestingly, the binding of mIFIT1 and hIFIT1 to the M40 RNA appears to generate more than one complex and generates complexes that are retained at the loading origin with increasing protein concentration; we speculate that these complexes represent the binding of multiple copies of IFIT1, with the binding perhaps being cooperative – the oligomerization of IFIT proteins has been characterized by other groups (reviewed in reference [59]). Even at the highest concentration of IFIT1 used here – 1,500 nM, the estimated peak cytosolic concentration of IFIT1 during a cellular antiviral response [60] – some amount of the P40 probe remained unbound, whereas the M40 probe was fully bound at concentrations well below 1,000 nM.

**Fig. 5.**
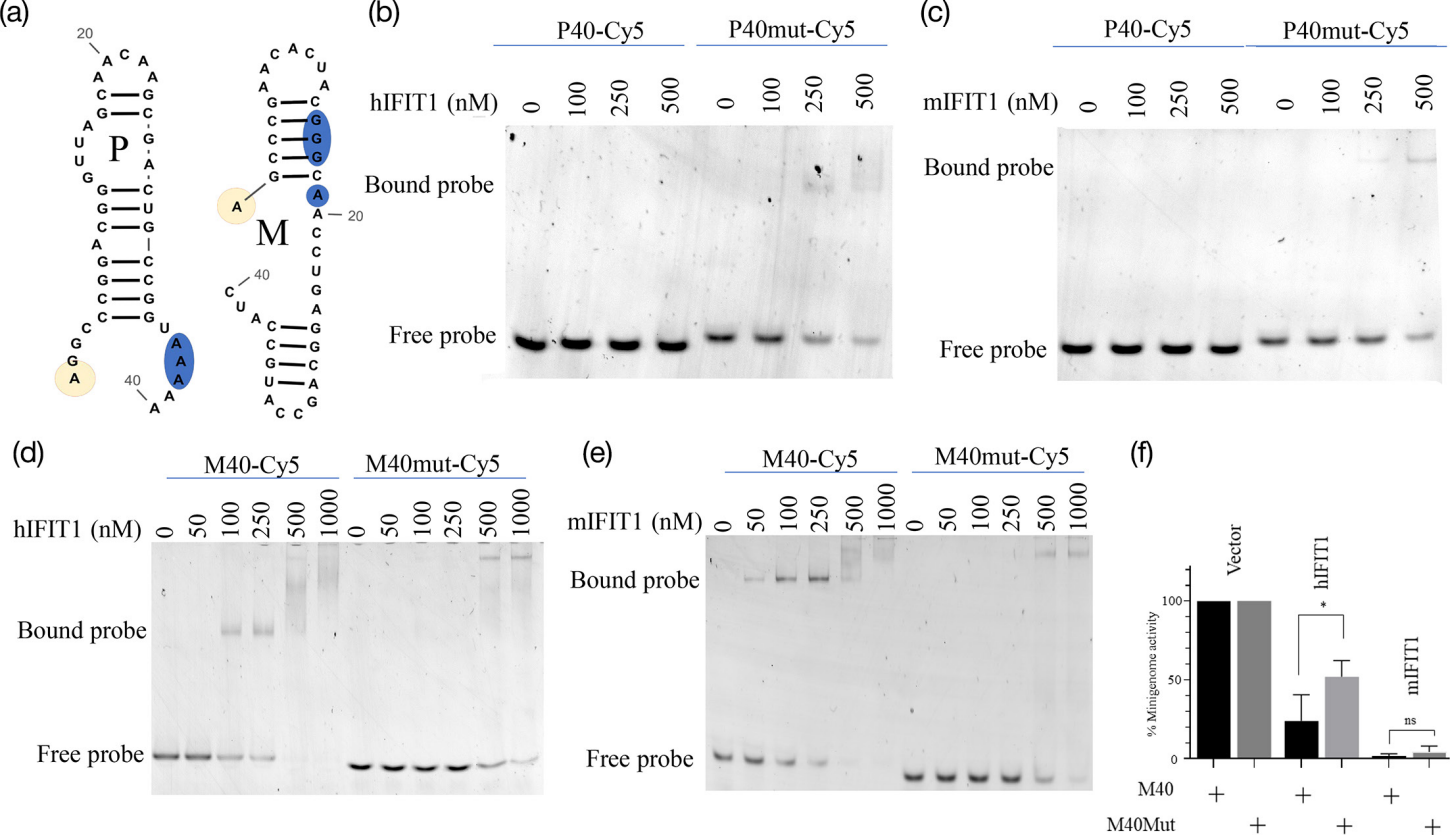
RNA secondary structure determines the relative affinity of PIV5 5′UTRs for IFIT1. (a) RNA secondary structure predictions of the mutated P40-mut and M40-mut sequences. The 5′ end is indicated by a yellow circle, and the altered bases are circled in blue. (**b–e**) Electrophoretic mobility shift assays with RNA probe added to a final concentration of 40 nM and the indicated concentration of purified recombinant IFIT1 protein: (**b**) P40 and P40-mut with hIFIT1, (**c**) P40 and P40-mut with mIFIT1, (**d**) M40 and M40-mut with hIFIT1 and (**e**) M40 and M40-mut with mIFIT1. (**f**) A bicistronic PIV5 minigenome (with the second cistron encoding firefly luciferase with either the M40 or the M40-mut 5′UTR) was co-transfected with the indicated IFIT1, and minigenome activity was assayed as before.

From the above data, it is not possible to exclude the possibility that the 5′-proximal sequence – rather than structure – of each UTR determines the affinity for IFIT1. Therefore, downstream mutations were designed, which alter the predicted structure of the P and M 5′UTRs without changing the RNA sequence at the 5′ end (Fig. 5a). Nucleotides 36–38 of P40 were mutated so that the new P40-mut sequence is predicted to lose the Cap-proximal secondary structure and form a four-base 5′-overhang, whilst nucleotides 15–17 and 19 of M40 were changed so that in the new M40-mut a hairpin is predicted to form, which leaves just a one-base 5′-overhang, matching the predicted 5′-overhangs of P and NP (see Fig. 4). To test whether the introduction of predicted 5′-terminal structure affects the affinity of the 5′UTRs for IFIT1, 40-base RNA probes of the new 5′UTRs were made and tested by electrophoretic mobility shift assay. Shifted complexes for P40-mut are present at protein concentrations at which P40 is not bound (<250 nM; Fig. 5b, c). For M40-mut, no protein is bound at low concentrations (<100 nM) in contrast to the unstructured M40 (Fig. 5d, e).

**Fig. 6.**
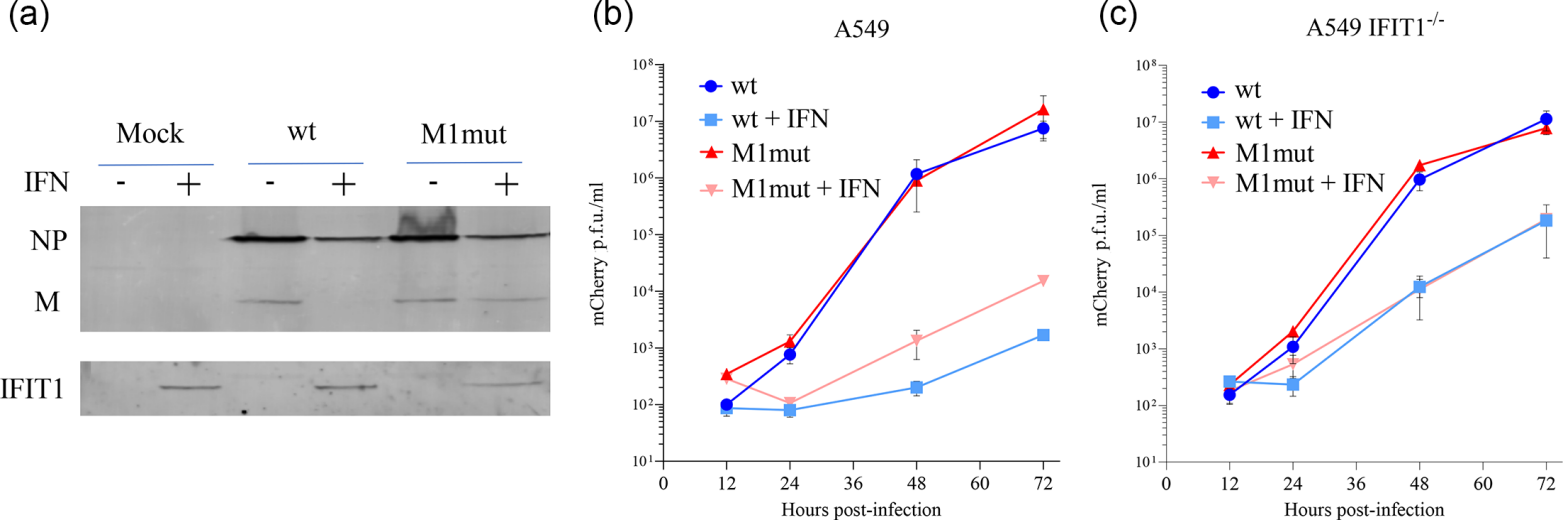
A structured M 5′UTR reduces the sensitivity of PIV5 to IFIT1. (**a**) A549 cells were or were not pre-treated with IFN for 8 h and then infected with the indicated virus at an moi of 5. Lysates were analysed by Western blot for NP, M and IFIT1 at 18 hpi. A549 (**b**) or A549 IFIT1^-/-^ (**c**) cells were or were not pre-treated with IFN for 8 h and then infected at an moi of 0.01 with either WT or M1 mutant virus. Virus samples were prepared at the indicated timepoints and titrated on Vero cells.

Next, we introduced these changes in the M 5′UTR into a bicistronic minigenome encoding firefly luciferase in the second cistron. The 5′UTR of the normal PIV5 minigenome cannot be altered due to the presence of the bipartite promoter, which is essential for replication [61,62], but the introduction of a second cistron allows for a comparison between M40 and M40-mut as 5′UTRs. Whilst both 5′UTRs were sensitive to IFIT1, M40-mut was significantly less sensitive to hIFIT1 than M40 (Fig. 5f). When we analysed the effects of mIFIT1, M40 and M40-mut both showed notable sensitivity; however, since mIFIT1 shows greater affinity for Cap0 than human IFIT1 [54], this result is unsurprising. Nevertheless, a small increase in the minigenome signal is seen with the M40-mut compared to the WT M40 when examined in the presence of mIFIT1. These data demonstrate that IFIT1 sensitivity and binding are not determined by the primary sequence of the first few bases but are rather dependent on the presence or absence of hairpin structures that leave a one-base overhang. These observations are consistent with the structure of IFIT1 bound to RNA in which the interactions between IFIT1 and the bound RNA are mainly with the inverted guanosine and the triphosphate bridge, and so, the primary sequence is not likely to affect IFIT1 binding in general [37]. Having established the mechanism by which PIV5 genes differ in their sensitivity to IFIT1, we wanted to see whether altering the IFIT1/IFN sensitivity of individual genes would alter the properties of the virus. To this end, we constructed a viral genome in which the 5′UTR of the M gene was changed (the same changes as used in the electrophoretic mobility shift assays discussed above) to render it less sensitive to IFIT1/IFN. The M1 5′UTR mutant virus (M1-mut) was rescued in BSRT7 cells and could be amplified in Vero cells. The incorporation of the mutation was confirmed by RT-PCR and sequencing with no evidence of reversion upon passage. When we examined the production of NP and M proteins in a high moi infection, we observed no difference in the level of NP and M proteins between WT and M1-mut viruses in the absence of IFN pre-treatment and thus the absence of IFIT1 (Fig. 6a). Upon induction of IFIT1 by IFN, the M protein was undetectable in the WT virus but could readily be detected in the M1-mut virus (Fig. 6a). The level of NP was reduced by IFIT1 by the same amount between viruses, showing that the difference is specific to the translation of M. Therefore, four base changes in the M 5′UTR, which introduce predicted 5′-proximal secondary structure, are sufficient to partially rescue translation of M from hIFIT1 during PIV5 infection.

The growth of the mutant virus was then compared to the WT virus on A549 cells and A549 cells lacking IFIT1. In the absence of IFN, both viruses grew to the same titre on A549 cells demonstrating that the M 5′UTR mutation has no attenuating effect and that it offers no advantage when IFN is absent (Fig. 6b, c). However, in IFN-pre-treated A549 cells, the mutant virus grew to an approximately tenfold greater titre than the WT virus by 72 hpi (Fig. 6b). Importantly, the growth advantage of the mutant virus over WT was lost in IFN-pre-treated A549 IFIT1^-/-^ cells (Fig. 6c).

These data show that PIV5 is sensitive to IFIT1 because it generates Cap0 mRNA and that, in the absence of fully methylated Cap structure, it is the presence or absence of 5′-proximal RNA secondary structure in the 5′UTRs of PIV5 genes that determines their relative sensitivity to IFIT1.

## Discussion

We previously identified IFIT1 as the ISG primarily responsible for the sensitivity of PIV5 to IFN. We had also previously observed that PIV5 transcripts from different genes were differentially sensitive to IFIT1, with the genes for the replicative components, NP and P, retaining some resistance to IFN/IFIT1, whilst the M transcript is fully sensitive. We had proposed that this resistance may reflect some degree of Cap1 methylation of IFIT1-resistant transcripts because Cap1 modification is sufficient to protect mRNAs against IFIT1 inhibition. Here, we show by CAP-MAP analysis that uninfected A549 cells do not contain Cap0 mRNA, whereas, upon infection, a significant level (20%) of transcripts have a Cap0 structure. Importantly, all of the Cap0 mRNA fraction in infected cells has an A residue at the first transcribed nucleotide; since all PIV5 transcripts begin with A, whereas greater than 50% of cellular mRNAs begin with G, all of the Cap0 mRNA would appear to be of viral origin. We have previously observed that viral transcripts comprise about 20% of the total mRNA in PIV5-infected cells [57], a number that is consistent with all of the viral mRNA being of Cap0 structure, although we cannot formally exclude a small fraction being of Cap1/2 structure; however, since there is no increase in the ratio of Cap1 mRNAs beginning with A residues upon infection, we think this is unlikely. The lack of Cap1-modified viral transcripts would explain the sensitivity of PIV5 to IFIT1. This likely also applies to other rubulaviruses such as MuV and PIV2, which are also sensitive to IFIT1 [25]. We and others have previously shown that non-*Rubulavirus* paramyxoviruses are not sensitive to IFIT1, suggesting that IFIT1 sensitivity may be unique to the *Rubulavirus* genus.

Structural comparisons of the L proteins of all members of *Mononegavirales* predict that they should all encode a 2′-O-MTase, which would generate the Cap1 structure. Despite the L protein of PIV5 containing a conserved MTase domain, it does not appear capable of generating fully Cap-methylated transcripts. Interestingly, although the alignment of domain VI of the L protein of various paramyxoviruses shows that the predicted 2′-O-MTase catalytic tetrad (K-D-K-E) is conserved across all genera, including *Rubulavirus*, there are numerous differences surrounding these residues, and it is clear that the *Rubulavirus* domain VI has diverged from that of non-*Rubulavirus* paramyxoviruses [63]. Perhaps the most notable difference is in the putative SAM-binding motif; the consensus sequence is GxGxG, whilst the *Rubulavirus* sequence is AxGxG. Whether these differences between *Rubulavirus* and non-*Rubulavirus* domain VIs affect the efficiency of 2′-O-methylation is not known. However, a systematic mutagenesis analysis of domain VI of Sendai virus by Murphy and Grdzelishvili [63] found that methylation efficiency is not affected by whether the SAM-binding motif is GxGxG or AxGxG, suggesting that an alanine in the first position of the motif does not prevent SAM-binding even if it may be suboptimal. Our data suggest that most – if not all – mRNA generated by PIV5 is Cap0. Therefore, we favour the explanation that the PIV5 2′-O-MTase is completely defective rather than simply less efficient. Together with the apparent divergence from non-*Rubulavirus* domain VIs, this makes it unlikely that single amino acid changes such as those in the SAM-binding motif could ‘restore’ 2′-O-MTase activity to PIV5. Instead, structural analyses such as that of Abdella *et al.* [64] may be necessary to determine whether the structure or position of the PIV5 MTase differs from the L proteins of paramyxoviruses with a functional 2′-O-MTase.

Given that rubulaviruses clearly have the coding capacity to express a functional 2′-O-MTase, the question arises as to what the functional significance might be for apparently losing this property. One possible explanation is that the highly efficient disruption of IFN signalling by the V protein reduces the pressure on PIV5 to generate Cap1 mRNA. However, the ~100-fold increase in virus titre seen in IFN-pre-treated cells when IFIT1 is knocked out (Fig. 1c) would suggest that there is a large fitness cost to the virus during infection. Instead, we propose that sensitivity to IFIT1 may counterintuitively be beneficial to PIV5 during persistent infections, particularly *in vivo* – as discussed below.

It is notable that the transcripts of the replicatory functions, NP, P and L, are predicted to have 5′-proximal secondary structure (albeit the structure of the L UTR is less stable than that of NP and P), whilst the mRNAs for structural genes (e.g. M) do not. We have shown here that this secondary structure is sufficient to limit the inhibitory effects of IFIT1. PIV5 is well-known for being able to establish persistent infections, and these infections are characterized by the formation of viral inclusion bodies [65]. These bodies are replication factories for the virus, containing NP, P and L proteins and viral RNA [66], and are not aggregates of precipitated proteins [67]. They are especially evident in cells that contain the virus in a persistent state, and we have demonstrated that such cells are able to serve as reservoirs of infectious virus for sustaining infection. Persistent infection would require that the virus be able to evade host immunity. In terms of innate immunity, PIV5 has the ability to block signalling in response to IFN and so should be able to evade the antiviral effects of IFN. However, in at least one case, a persistent infection by PIV5 has been shown to be associated with a V protein that cannot block IFN signalling [13,68], which would potentially leave the virus vulnerable. It is tempting to speculate that the combination of a 2′-O-MTase-deficient L protein and the evolution of a subset of 5′UTRs that are IFIT1-resistant arms the virus with a mechanism to maintain its replicative functions despite an active innate immune response. Interestingly, an IFIT1-sensitive strain of the alphavirus VEEV is able to form persistent infections in cell culture in which the level of IFIT1 correlates with control of the virus [69]. Downregulation of viral translation *in vivo* could limit the ability of the adaptive immune response to recognize cells in an IFN-induced antiviral state that have been infected with PIV5 and that may become persistently infected. Thus, lack of expression of M, HN and F will limit the number of T-cell epitopes available for recognition by cell-mediated immune responses. In this regard, it is of note that the dominant PIV5 Cytotoxic T Lymphocyte epitope in BALB/c mice is on the M protein [70]. Furthermore, infected cells, which do not express the HN or F proteins on their surface, will not be killed by antibody-mediated cell cytotoxicity. Also, the M protein, whose expression is highly sensitive to IFIT1, plays a critical role in anchoring the HN and F proteins to the cell surface, and, in the absence of M, both HN and F have a very short half-life. Interestingly, the *Rhabdoviridae* M protein has been linked to CPE in infected cells [71,72], meaning that suppression of M translation could prolong the life of the infected cell. Within *Paramyxoviridae*, persistence of Sendai virus has been linked to temperature-sensitive mutations in M [73,75], and most notably, the persistence of MV in the brain of individuals with sub-acute sclerosing panencephalitis (SSPE) is linked to the loss of M expression due to hypermutation [76,81], suggesting that the absence of M may be a prerequisite for persistence of MV [82,83]. Whilst the loss of M in cases of SSPE is irreversible and thus unlikely to be a successful viral persistence strategy for spread to new hosts, the reversibility of suppression of PIV5 M translation by IFIT1 presents a possible mechanism for maintaining virus persistence whilst allowing for reactivation of particle production when IFIT1 levels decrease.

We are struck by our observation that, whilst each of the IFIT1-resistant transcripts has a short region of secondary structure proximal to the 5′ Cap, each of these structures retains a single unpaired base at the extreme 5′ end. We speculate that this feature has evolved to avoid the inadvertent activation of RIG-I, which has been reported to bind to Cap0 mRNA when the 5′-terminal base is paired [84,85], but cannot do this when the 5′UTRs have an unpaired one-base 5′-overhang. Thus, the structured viral transcripts would be resistant to IFIT1 but would not act as a PAMP for RIG-I.

Although *in vitro* cell culture experiments can elucidate ways in which the viruses have evolved to downregulate RNA synthesis or translation, *in vivo* models are needed to prove the benefits to the virus in terms of long-term persistence as opposed to short-term acute replication. Further study of the interactions between PIV5 and the IFN system should increase our understanding of ISGs such as IFIT1 and possibly provide insight into the mechanisms by which paramyxoviruses are able to form persistent infections.

## Abbreviations

CAP-MAP: cap analysis protocol with minimal analyte processing
CMTR: Cap methyltransferase
CRISPR: clustered regularly interspaced short pallindromic repeats
DMEM: Dulbecco’s Modified Eagle Medium
F: fusion
HN: haemagglutinin-neuraminidase
ISGs: IFN-stimulated genes
L: large
LC: liquid chromatography
M: matrix
m7G: N7-methylated guanosine
mIFIT1: murine IFIT1
moi: multiplicity of infection
MuV: mumps virus
MV: measles virus
NP: nucleoprotein
2′-O-MTases: 2′-O-methyltransferases
PEI: polyethyleneimine
p.f.u.: plaque forming units
PIV5: parainfluenza virus 5
SSPE: sub-acute sclerosing panencephalitis
VEEV: Venezuelan equine encephalitis virus
